# Neutrophil Elastase Induces Chondrocyte Apoptosis and Facilitates the Occurrence of Osteoarthritis *via* Caspase Signaling Pathway

**DOI:** 10.3389/fphar.2021.666162

**Published:** 2021-04-14

**Authors:** Ganyu Wang, Weiqiang Jing, Yuxuan Bi, Yue Li, Liang Ma, Hui Yang, Yuankai Zhang

**Affiliations:** ^1^ Department of Pediatric Surgery, Qilu Hospital, Cheeloo College of Medicine, Shandong University, Jinan, China; ^2^ Department of Urology, Qilu Hospital, Cheeloo College of Medicine, Shandong University, Jinan, China; ^3^ Shandong Provincial Key Laboratory of Infection and Immunology, Department of Immunology, School of Basic Medical Sciences, Cheeloo College of Medicine, Shandong University, Jinan, China; ^4^ Department of General Surgery, Shandong Qianfoshan Hospital, Cheeloo College of Medicine, Shandong University, Jinan, China; ^5^ Department of Orthopedics, Qilu Hospital, Cheeloo College of Medicine, Shandong University, Jinan, China; ^6^ Department of Radiology, Qilu Hospital, Cheeloo College of Medicine, Shandong University, Jinan, China

**Keywords:** osteoarthritis, neutrophil elastase, chondrocyte, apoptosis, caspase-3

## Abstract

Osteoarthritis (OA) is the most common and prevalent chronic joint disorders in the elderly population across the globe, resulting in severe disability and impairment of quality of life. Existing treatment can only alleviate the symptoms and delay the progression of OA. Therefore, novel and effective therapeutics strategies for OA need to be developed. Our present study first found that neutrophil elastase (NE) was significantly increased in OA patients’ synovial fluid. Next, we examined the effect of neutrophil elastase (NE) on chondrocytes *in vitro* and *in vivo*. The results showed that NE suppressed cell proliferation, induced apoptosis and prevented cell migration in chondrocytes *in vitro*, accompanied by the elevation of intracellular ROS and calcium level. Moreover, NE enhanced the cleaved caspase-3 levels and disrupted the mitochondrial transmembrane potential balance. Meanwhile, chondrocytes apoptosis induced by NE can be alleviated by caspase inhibitor, zVAD-FMK and antioxidants, GSH. Besides, treatment of sivelestat, the inhibitor of NE, significantly reduced the pathological processes in OA model rats *in vivo*. The results of our study suggested that NE is an important factor in OA, which induces chondrocyte apoptosis and facilitates the occurrence of OA *via* caspase signaling pathway, and targeting the crucial signal centering around NE may be the potential therapies for OA.

## Introduction

Osteoarthritis (OA), the most prevalent degenerative disease of the articulating joints, which is characterized by changes in chemical-physico properties of synovial fluid, articular cartilage degradation, subchondral bone remodeling and pain, leads to a major cause of chronic disability and significant impairment of life quality in elderly populations (Bijlsma et al., 2011; Cross et al., 2014; Glyn-Jones et al., 2015; Palazzo et al., 2016). Due to the growing aging of population in developing and developed countries, as well as the increasing risk factors of OA, especially the increasing prevalence of obesity and a sedentary lifestyle, the prevalence of OA in humans has been continuously increasing (Litwic et al., 2013; Sacitharan, 2019). Medical care was used for alleviating the clinical symptoms and delaying the progression of OA, using physiotherapy, analgesics, intra-articular steroid injection, oral supplementation, and ultimately ends with joint replacement surgery (Felson, 2006; Arden et al., 2020).

Adult articular cartilage is a highly organized dense connective tissue that covers the articulating ends in synovial joints and provides low friction during joint movement. Extracellular matrix (ECM), a highly dynamic structure, that synthesized by chondrocytes provides strength and tension in articular cartilage (Goldring 2012). The survival and function of chondrocytes are important for the homeostasis of articular cartilage, and osteoarthritis is characterized by chondrocyte death that leading to extracellular matrix (ECM) damage and articular cartilage destruction (Hwang and Kim, 2015; Charlier et al., 2016). Chondrocytes cannot regulate the structure and function of articular cartilage during they undergo death that cannot produce enough extracellular matrix for consumption (Kim et al., 2000).

OA pathophysiology was supposed to biomechanical constraint exerted on weight-bearing joints. The metabolic and genetic factors are involved in the complex multifactorial pathogenesis of OA. It is becoming increasingly evident that multiple inflammatory mediators were released into the synovial fluid of joint that serve an important role in initiation and progression of synovial inflammation and articular cartilage degradation (Kapoor et al., 2011). Multiple of inflammatory cells, such as macrophages, neutrophils and lymphocytes, are recruited into the site of injury and produce catabolic and proinflammatory mediators to initiate tissue repair. Serine proteases released by recruited immune cells in inflammatory sites, such as neutrophil elastase (NE), protease 3 (PR3), trypsin and cathepsin G (CG), contribute to the damage of articular cartilage and subchondral bone remodeling (Korkmaz et al., 2010). In the pathological states of OA, macrophages firstly migrate to the inflammatory sites and alter their phenotype to mediate tissue repair. In addition, the injured tissue and cells can release pathogen- or damage-associated molecular patterns (PAMPs or DAMPS) which activate inflammatory signaling pathway in macrophages to release various cytokines and further recruit other inflammatory cells, like neutrophils, to the injured site. Neutrophils, as a type of polymorphonuclear leukocyte, are typically recognized as the first leukocytes to be recruited to the inflammatory site and form the earliest line of defense against invading microorganisms. NE is a major type of neutrophil serine proteases (NSPs) stored in the cytoplasmic blue granules of neutrophils. Combined with reactive oxygen species (ROS), it helps to degrade the phagocytic microorganisms in the phagocytic lysosomes, and thus helps to regulate inflammation and immune response.

Apoptosis is a kind of programmed cell death which is finely regulated and related to the specific biochemical and morphological changes of cells (Elmore, 2007). Dysregulation occurs in apoptotic pathways are implicated in various pathological conditions including cancer, dysplasia, degenerative diseases and immune disorders (D'Arcy, 2019). Apoptosis is activated by three main pathways: the intrinsic apoptotic pathway (mitochondrial pathway), extrinsic apoptotic pathway (death receptor pathway) and perforin/granzyme pathway. In these apoptosis pathways, caspase-3 plays a dominant role by mediating programmed cell death through various apoptotic signaling pathway.

Caspases are a specific group of cysteine aspartic proteases in the processes of apoptosis, which are divided into initiators of apoptosis (caspases 2, 8, 9, and 10), executioner of apoptosis (caspases 3, 6, and 7) and inflammatory caspases (caspases 1, 4, 5, 11, and 12). Apoptotic caspases are known to regulate apoptosis through intrinsic and extrinsic pathways. Caspase-3 is the most common executioner caspase, and activated caspase-3 is responsible for the cleavage of numerous substrates, inducing apoptosis by activating a series of signaling pathways, including DNA fragmentation, cytoskeleton reconstruction, formation of apoptotic bodies, phosphatidylserine (PS) eversion to the outside of cell membrane and so on. Finally, apoptotic cells are recognized and phagocytized by phagocytes (Medina et al., 2020). In all caspase family members, caspase-3 is at the core of caspase cascade, and is activated by both extrinsic and intrinsic apoptotic pathways during apoptosis. Many diseases, including tumor, infection, autoimmune diseases, neurodegenerative diseases and immunodeficiency, can be caused by abnormal activation or inactivation of caspase (Li and Yuan, 2008).

Current treatments for OA only provide short-term benefit, but cannot prevent or cure OA by non-surgical methods, and most patients with OA use diverse pharmacological agents, including NSAIDs, IAI of corticosteroid or HA. Because OA is a progressive disease, clinicians and scientists have been looking for an ideal drug to prevent the destruction of articular cartilage, which can be well tolerated and effectively delay the progress of OA. A variety of signaling pathways in apoptosis pathway provide many potential targets for the protection of articular chondrocytes. Since chondrocyte apoptosis is an important cellular mechanism of human OA, blocking chondrocyte apoptosis may be an effective strategy to prevent cartilage degeneration.

Therefore, our study aims to determine whether NE can induce chondrocyte apoptosis through caspase signaling pathway and participate in the occurrence and development of OA through *in vitro* and *in vivo* experiments. Through this study, we hypothesize that inhibition of NE activity or abnormal activation of caspase signaling pathway in OA can delay the progress and alleviate the pathological changes of OA.

## Materials and Methods

### Materials

DMEM/F-12 medium was purchased from MACGENE. Fetal Bovine Serum (FBS) were purchased from Corning. Neutrophil elastase, Propidium Iodide (PI), sulforhodamine B (SRB), Rat IgG and RNase A were purchased from Solarbio. N-methoxysuccinyl-Ala-Ala-Pro-Val p-nitroaniline was provided by Beijing Hvsf United Chemical materials company. FITC AnnexinV Apoptosis Detection Kit were purchased from BD Bioscience. Triton X-100 solution, Mitochondrial membrane potential assay kit using JC-1, Ca^2+^ fluorescent probe Fluo-4/AM, GreenNuc™ Caspase-3 assay kit for Live Cells, One Step TUNEL Apoptosis Assay Kit and Reactive Oxygen Species (ROS) Assay Kit were purchased from Beyotime. Western blot primary antibody Diluent was purchased from SparkJade. BCA protein assay kit was purchased from Thermo Fisher Scientific. Western Bright™ ECL-Plus was purchased from EMD Millipore. DAPI, phalloidin and D-Luciferin sodium salt was purchased from YEASEN.

### Patient Samples

OA cartilage tissues and synovial fluid were obtained from patients who underwent total knee or hip arthroplasty and normal cartilage tissues were collected from patients with ligament injury and undergoing ligament reconstruction surgery at Qilu Hospital of Shandong University. OA was diagnosed by clinical history, detection and combined with clinical imaging examination, in the meantime, gross pathological assessment was performed during joint replacement. Patient with ligamentous injury had no joint disease and no abnormalities of cartilage. Studies involving human specimens have been approved by the Ethical Committee of the Qilu Hospital of Shandong University (Jinan, China), and all tissue samples were obtained with informed consent from all patients.

### NE Activity Assay

The synovial fluid samples for the assay of NE activity were preserved in liquid nitrogen before analysis. N-methoxysuccinyl-Ala-Ala-Pro-Val p-nitroaniline (produced by Beijing Hvsf United Chemical materials company, Beijing, China) was used for the substrate of NE. The hydrolytic activity of the substrate was measured spectrophotometrically. 1 mM substrate was added to 0.1 ml synovial fluid sample in 0.1 M Tris HCl buffer (pH = 8.0) containing 0.5 M NaCl and reached a total volume of 1.0 ml at 25°C. After the reaction system was incubated at 37°C for 24 h, the absorbance value was measured at 405 nm by a microplate reader (Yoshimura et al., 1994).

### Culture of Chondrocytes

The human chondrocyte cell line used in this study is C-28/I2, which was provided by American Type Culture Collection (ATCC). C-28/I2 cells were incubated in Dulbecco's Modified Eagle Medium: F-12 (DMEM/F-12) with 10% fetal bovine serum (FBS), 100 U/ml penicillin-streptomycin, and the culture dishes were placed in a 5% CO_2_ incubator at 37°C. For use in the experiments, cells were harvested using trypsin, washed twice with DMEM/F-12, and cultured in six-well culture plate at 1 × 10^5^ cells/well. At confluence of 50%, cells were cultured under different doses of NE (cat., no. E8210; Solarbio) overnight.

### Cell Proliferation Assay

The anti-proliferative effect of NE on chondrocytes was examined by Sulforhodamine B (SRB) assay according to the manufacturer’s instructions (Orellana and Kasinski, 2016). Briefly, chondrocytes were seeded at a density of 2 × 10^3^ cells per well in 96-well plates. Chondrocytes were treated with NE (1, 2, 5, 10 U/mL) 12 h after seeding. At 24 h, 48 h, 72 h time points, chondrocytes were washed twice with PBS followed by one hour fixation using 10% trichloroacetic acid. After fixation, trichloroacetic acid was aspired followed by twice washes with PBS and thereafter stained with 0.4% SRB dissolve in 1% acetic acid solution for 15 min. Then staining solution was discarded and plates were washed using 1% acetic acid and allowed to dry overnight. The dye was solubilized in 10 mM Tris base solution and the optical density (OD) value at 510 nm was read and analyzed on a microplate reader.

### Annexin V-Fluorescein Isothiocyanate (FITC)/PI Apoptosis Assay

Chondrocytes were seeded with a density of 1 × 10^5^ cells per well in 6-well plates. After chondrocytes were treated as indicated, cells were harvested. The FITC-AnnexinV/PI Apoptosis Detection Kit (cat., no. 556547; BD Biosciences) was used to access apoptosis in each group of chondrocytes according to the manufacturer’s instructions. After treated with NE for 48 h, cells were collected and then suspended in 500 μl binding buffer containing 5 μl of annexin V-FITC (20 μg/ml) and 15 μl of PI (50 μg/ml). Cell apoptotic rate (%) were detected by flow cytometry following 20 min of incubation at room temperature.

### Caspase-3 Assay

A GreenNuc™ Caspase-3 assay kit for Live Cells (cat. no., C1168S; Beyotime Institute of Biotechnology) was used to measure caspase-3 activity, according to kit instructions. After treated with NE for 24 h, single-cell suspensions were collected and added in 1 μl GreenNuc™ Caspase-3 substrate, mixed thoroughly and incubated for 20 min at 37°C in the dark. The levels of caspase-3 activity were determined by flow cytometry (Crowley and Waterhouse, 2016).

### Terminal Deoxynucleotidyl Transferase dUTP Nick End Labeling Analysis

To detect the apoptotic levels of chondrocytes, the cell death detection kit based on the TdT-mediated dUTP nick end labeling (TUNEL) to detect DNA fragmentation was used. After treated with NE for 24 h, cells were trypsinized, pelleted and washed twice. The supernatant was removed and cells were fixed with 500 µl 4% paraformaldehyde solution for at least an hour at 4°C, followed by centrifugation to remove fixative solution. Subsequently, cells were washed twice with PBS and resuspended in 0.1% Triton X-100 for 10 min on ice before being washed twice with PBS and resuspended in 50 µl TUNEL reaction mixture. Cells were incubated for 30 min at 37°C in the dark, and the apoptotic cells were quantified by flow cytometry (Majtnerová and Roušar, 2018).

### Intracellular Calcium Determination

For the measurement of intracellular calcium levels in chondrocytes, cells were dissociated with trypsin after treated with NE for 24 h and calcium levels were determined by using a calcium sensitive dye, Fluo-4/AM (cat. no., S1060; Beyotime Institute of Biotechnology). Cells were treated with NE (1, 2, 5, 10 U/ml) for 24 h and washed twice with PBS. Subsequently, cells were incubated with Fluo-4/AM for 30 min at 37°C, then washed with PBS twice to remove the uncombined Fluo-4/AM. The fluorescence intensity was measured by flow cytometry.

### Analysis of Mitochondrial Membrane Potential

The mitochondrial membrane potential was detected by flow cytometry using a JC-1 mitochondrial membrane potential assay kit (cat. no., C2006; Beyotime Institute of Biotechnology). After treated with NE for 24 h, the cells were collected, washed twice in PBS to remove the cell media and incubated with JC-1 for 20 min at 37°C. The cells were then rinsed twice with 1× JC-1 staining buffer and the fluorescence intensity was analyzed by flow cytometry.

### Detection of Reactive Oxygen Species

The intracellular ROS levels were measured using a Reactive Oxygen Species Assay Kit (cat. no., S0033; Beyotime). After treated with NE for 24 h, chondrocytes were incubated with 5 μM dichlorofluorescein diacetate (DCFDA) at 37°C for 30 min. Then cells were washed twice with PBS and intracellular ROS production were immediately analyzed with flow cytometry, in accordance with the instructions from manufacturer.

### Wound-Healing Assay

Tissue repair capacity of chondrocytes was evaluated by wound-healing assay. A single scratch wound was made using a 200 μl sterile pipette tip on a confluent monolayer. After washed twice with PBS, the cells were cultured with different doses of NE (1 and 2 U/ml). Then the scratch wounds were captured at 0 h, 12 h, and 24 h after scratching with an inverted microscope.

### Confocal Microscope

The cultured chondrocytes were inoculated into confocal culture dishes at a density of 1 × 10^5^/well and cultured for 24 h. Then the cells were treated with various concentrations of NE (1 and 2 U/ml). Cell cultures were then fixed with 4% paraformaldehyde at 4°C respectively. Subsequently, the nuclei were labeled with DAPI (in blue), and microtubule was labeled with phalloidin conjugated to TRITC (in red) (YEASEN, cat# 40734ES75) using standard protocols. A ZEISS high sensitivity laser confocal microscope was used to obtain the fluorescence image.

### OA Models

All the animal experimental procedures have obtained the approval from the Ethical Committee of the Qilu Hospital of Shandong University (Jinan, China). All 10-week-old specific pathogen-free (SPF) Sprague-Dawley (S-D) rats (220–230 g in weight) were purchased from the Laboratory Animal Center of the Shandong University. Total rats were randomized into three groups: sham group, OA model group and treatment group. Rats were anesthetized (pentobarbital sodium; 0.3%, 0.1 ml/10 g), and the plane of anesthesia was accessed by a withdrawal reflex. Experimental OA was surgically induced in the right knee joint according to intra-articular injection of NE (20 U/kg). The rats in model group and treatment group were respectively received an injection of normal saline or NE inhibitor sivelestat (50 mg/kg, i.p) once daily postoperatively. In the sham group, only the skin of the right knee joint was resected and normal saline was injected into articular cavity. After the treatment of sivelestat for 2 weeks, the changes of joint space and articular cartilage calcification in each group were evaluated using the Micro-computed tomography (Micro-CT) imaging (Liu et al., 2019). Knee joints were harvested 2 weeks later.

### Histological Assay

Cartilage destruction induced by NE was assessed using Safranin O-fast green staining. At the end of the animal experiment, the knee joints of rats in different groups were obtained. The whole knee joints were dissected and fixed in 4% polyformaldehyde for 24 h and then decalcified with 0.5 M ethylenediaminetetraacetic acid (EDTA). After routine dehydrated, transparent and paraffin embedded, the joints were sliced into 5 μm continuous sections. The articular cartilage degeneration in knee joint was accessed using Safranin O-fast green staining in accordance with the standard procedures. The histomorphometric analysis of the cartilage was analyzed by an Osteoarthritis Research Society International (OARSI) scoring system (Gerwin et al., 2010; Zhou et al., 2016).

### Western Blotting Analysis

The collected samples were added in ice-cold RIPA lysis buffer containing protease and phosphatase inhibitor mixture, and the total protein content was quantified with a BCA protein assay kit (cat. no., NCI3227CH; Thermo Fisher Scientific, Inc.). Equivalent amounts of proteins were separated by 10% SDS-PAGE and transferred onto poly-vinylidene difluoride (PVDF) membranes. The membrane was blocked in 5% skim milk and then incubated with the following primary antibodies overnight at 4°C: Cleaved caspase-3 (D175) (5A1E) Rabbit mAb (CST, cat# 9,664), Beta-Actin (13E5) Rabbit mAb (CST, cat# 4,970), Caspase-3 (D3R6Y) Rabbit mAb (CST, cat# 14,220), Phospho-p44/42 MAPK (Erk1/2) (Thr202/Tyr204) (D13.14.4E) XP^®^ Rabbit mAb (CST, cat# 4,370), p44/42 MAPK (Erk1/2) (137F5) Rabbit mAb (CST, cat# 4,695). After washing with TBST, the membranes were incubated with horseradish peroxidase (HRP)-conjugated secondary antibody for 1 h at room temperature. The proteins bands were detected with an ECL Detection kits (cat. no., WBKLS0050; EMD Millipore).

### Statistical Analysis

We performed at least three times replications in each reported experiment. Data analysis was performed using Prism software (GraphPad Prism version 7.0; GraphPad Software, Inc.). Statistical analyses were performed using one-way ANOVA. Error bars for SEM are shown. Where indicated in the figures, degrees of *p*-value significance are as follows: *p < 0.05, **p < 0.01 and ***p < 0.001.

## Results

### NE was Overexpressed in OA Patients

**FIGURE 1:**
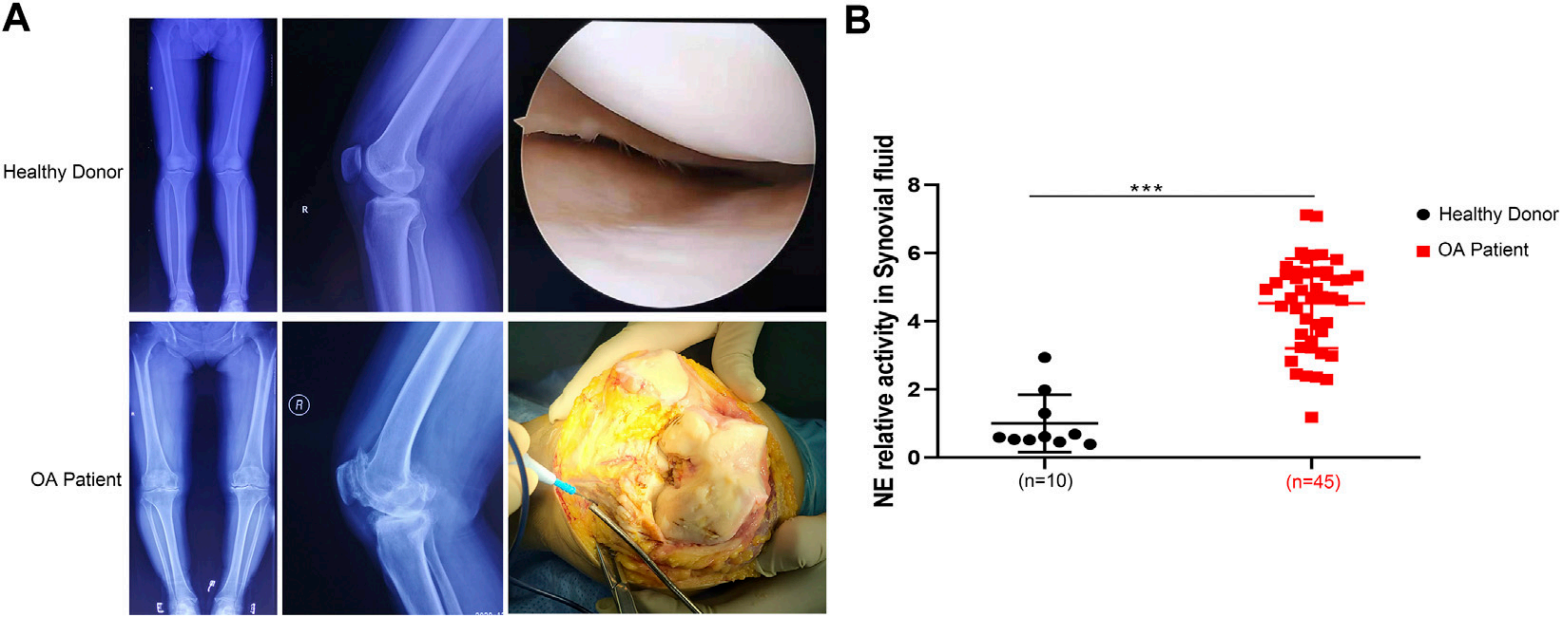
Neutrophil elastase is overexpressed in OA patients. **(A)** X ray images and macroscopic views (arthroscopic image and intraoperative image) of knee joint from healthy donors and OA patients. **(B)** NE relative activity in synovial fluid of healthy donors and OA patients. Data are mean ± SEM; *n* = 10 (healthy donors), *n* = 45 (OA patients). ***p < 0.001.

We performed radiological evaluations on knee joints from different groups of patients to verify typical imaging manifestations of OA. The results showed that preoperative knee X-ray and gross image from OA patients revealed bone spurs, subchondral sclerosis, and a narrowed joint space caused by OA compared with that from healthy donor (Figure 1A). To explore the differences between the two types of synovial fluid, we examined the concentrations of a variety of proteins and cytokines. The results showed that the expression of NE in OA patients was significantly higher than that in non-OA patients (Figure 1B), indicating that NE may be involved in the pathogenesis of OA.

### NE Suppressed Cell Proliferation and Accelerated Apoptosis of Chondrocytes

**FIGURE 2:**
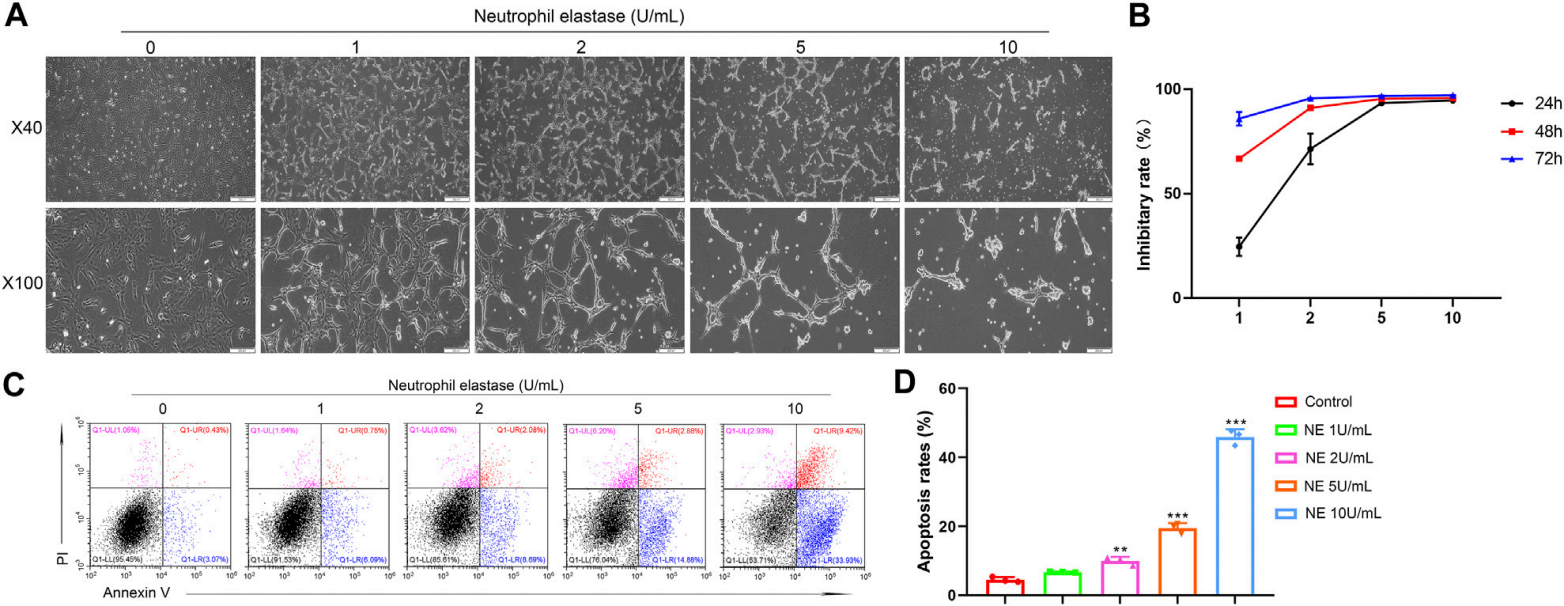
Neutrophil elastase shows potent growth inhibition in chondrocytes *in vitro.* **(A)** Representative images of chondrocytes treated with NE for 48 h. The representative fields were photographed at ×40 and ×100 magnification. **(B)** Inhibitory rate histogram of chondrocytes treated with NE for 24 h, 48 h, and 72 h. **(C)** The fluorescence pattern of annexin V-FITC/PI staining chondrocytes*.* **(D)** Percentages of annexin V positive cells for chondrocytes treated with different doses of NE. Data are mean ± SEM; *n* = 3. **p < 0.01, ***p < 0.001.

Next, a collection of *in vitro* and *in vivo* experiments helped us to explore how NE affects OA and the intrinsic mechanisms involved. It is widely accepted that apoptosis is a multi-step process of programmed cell death occurring in many cellular systems. Apoptotic cells undergo characteristic morphological changes, which include cell shrinkage, nuclear condensation, DNA fragmentation, and so on (Elmore, 2007). As shown in Figure 2A, we took photos of chondrocytes after treating with different doses of NE (1, 2, 5, 10 U/ml) for 48 h. Compared with the control groups, sparse shrinkage spindle-shaped chondrocytes and worse cell adhesion were observed in NE-treated group.

The effect of NE on chondrocytes proliferation was analyzed at different concentrations (1, 2, 5, 10 U/ml) for 24 h, 48 h, and 72 h using SRB assay. As shown in Figure 2B, NE reduced the viability of chondrocytes markedly. These results revealed that cellular proliferation was impeded by NE in a dose and time-dependent manner.

Accumulating evidence exists suggesting that OA markedly associates with chondrocyte apoptosis. Therefore, to test whether NE exert apoptotic effect on chondrocytes, we treated chondrocytes with different doses of NE (1, 2, 5, 10 U/ml), and the apoptosis rate of chondrocytes was determined by Annexin V-FITC/PI staining. As shown in Figures 2C,D, flow cytometric analysis revealed that there is a significantly increase in the percentage of apoptotic cells from NE-treated group compared with control group and the chondrocytes exhibited a remarkably dose-responsive increase of apoptotic fractions in NE-treated cultures for 48 h. As a whole, these results indicated that NE can induce chondrocytes apoptosis which facilitates articular cartilage degeneration in OA *in vitro*.

### NE Induces Chondrocytes Apoptosis Through a Caspase-Dependent Pathway

**FIGURE 3:**
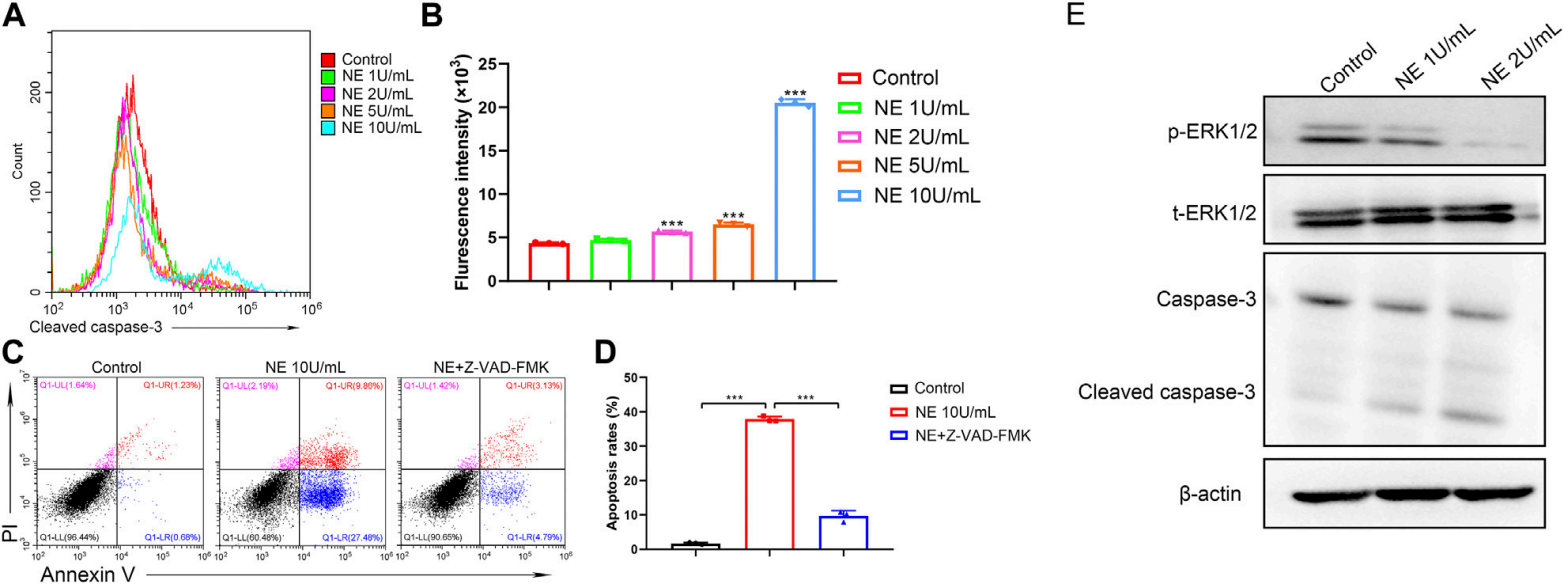
Neutrophil elastase induces chondrocytes apoptosis through cleaved caspase-3*.* **(A)** Representative histograms of cleaved caspase-3 levels of chondrocytes were shown. **(B)** Cell associated mean relative fluorescence intensities. Data are mean ± SEM; *n* = 3. ***p < 0.001. **(C)** The fluorescence pattern of annexin V-FITC/PI staining chondrocytes. **(D)** Percentages of annexin V positive cells for chondrocytes treated with NE and Z-VAD-FMK. Data are mean ± SEM; *n* = 3. ***p < 0.001. **(E)** Western blot of ERK1/2 phosphorylation, total ERK1/2, caspase-3 and cleaved caspase-3 levels of chondrocytes treated with NE.

Caspases are expressed intracellular cysteine proteases that mediate cell death and inflammation process. Specifically, caspase-3 is an indispensable mediator in both apoptotic and necrotic cell death. To further investigate the intrinsic apoptotic pathway in chondrocytes induced by NE, we use flow cytometry to detect the levels of cleaved caspase-3. As shown in Figures 3A,B, the chondrocytes exhibited significantly higher levels of cleaved caspase-3 following NE treatment. Then chondrocytes were treated with N-benzyloxycarbonyl-valyl-alanyl-aspartyl-fluoromethyl ketone (Z-VAD-FMK, 20 μM), a cell-permeable and irreversible pan-caspase inhibitor, which can inhibit the activation of caspase-3 in chondrocytes. The results in Figures 3C,D indicated that Z-VAD-FMK can protect the chondrocytes from apoptosis induced by NE. Meanwhile, we measured the levels of caspase-related protein by western blot. As shown in Figure 3E, NE treatment led to an increase in cleaved caspase-3 levels, and a decrease in the phosphorylation of ERK1/2 levels in a dose-responsive manner. These results demonstrated that the pro-apoptotic effect of NE in chondrocytes is owing to caspase-3 activation.

### NE Induces Chondrocytes DNA Degradation

**FIGURE 4:**
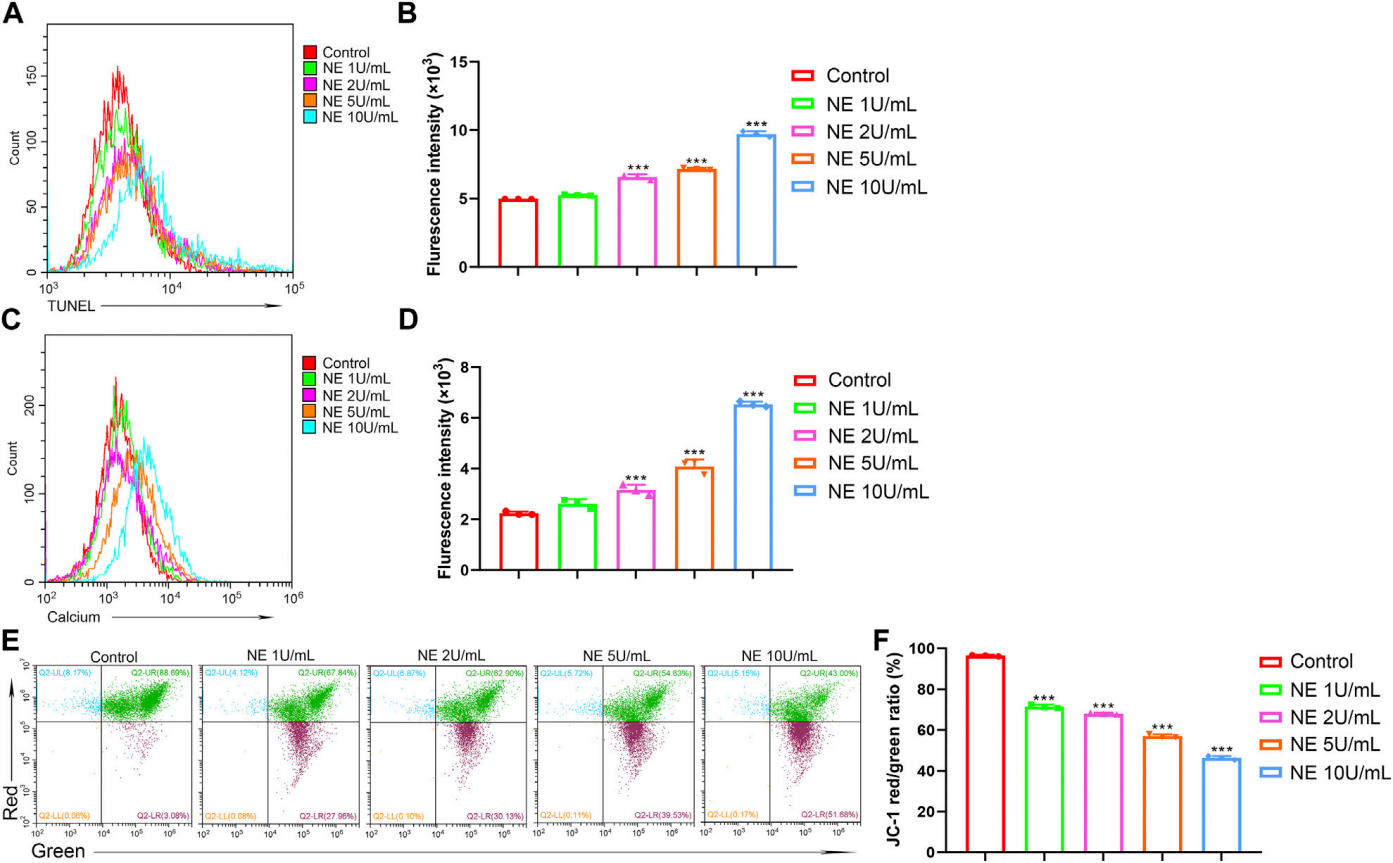
Neutrophil elastase induces DNA fragmentation, mitochondrial disruption and intracellular calcium release of chondrocytes. **(A,B)** TUNEL levels of chondrocytes were measured by flow cytometry. **(A)** Representative histograms were shown. **(B)** Cell associated mean relative fluorescence intensities. Data are mean ± SEM; *n* = 3. ***p < 0.001. **(C,D)** Intracellular free Ca^2+^ of chondrocytes were measured by flow cytometry. **(C)** Representative histograms were shown. **(D)** Cell associated mean relative fluorescence intensities. Data are mean ± SEM; *n* = 3. ***p < 0.001. **(E,F)** JC-1 staining of chondrocytes were measured by flow cytometry. **(E)** The fluorescence pattern of JC-1 staining chondrocytes. **(F)** Red/Green fluorescence intensities. Data are mean ± SEM; *n* = 3. ***p < 0.001.

Degradation of DNA by endonucleases into oligonucleosomal DNA fragments is considered as the biochemical hallmark of apoptosis. DNA degradation can be detected by TUNEL assay, which relies on the identified labeling to a 3′-hydroxyl termini of DNA break ends by TdT (Majtnerová and Roušar, 2018). To evaluate the level of DNA degradation in chondrocytes with NE treatment, TUNEL fluorescence intensity was measured by flow cytometry. As shown in Figures 4A,B, NE-treated chondrocytes indicated a high TUNEL fluorescence intensity vs. the controls, which revealed that the NE-induced apoptosis in chondrocytes was connected with DNA degradation.

### NE Induces Intracellular Free Ca^2+^ Elevation in Chondrocytes

Accumulating evidence suggests that calcium serves as a major intracellular second messenger in cellular physiology and play a critical role in cell apoptosis. Sustained elevation of Ca^2+^ in cytoplasm can result in Ca^2+^ homeostasis disruption and finally trigger cell apoptosis (Pinton et al., 2008). To investigate the role of calcium signaling in NE-induced apoptosis of chondrocytes, we used calcium indicator dye Fluo-4/AM to monitor the levels of intracellular cytosolic Ca^2+^. As shown in Figures 4C,D, the fluorescence intensity from the NE-treated group was markedly higher than the control group, which indicated that NE treatment resulted in elevated levels of Ca^2+^ in the cytoplasm of chondrocytes. These results showed that NE-induced chondrocyte apoptosis is associated with the disruption of the intracellular calcium balance.

### NE Disrupts the Mitochondrial Membrane Potential of Chondrocytes

Mitochondria, known as the power houses of the cell, play a crucial part in the physiological activity. The electron transport chain controls ATP synthesis and generates the mitochondrial membrane potential (ΔΨ), which is reflective of the functional metabolic state of mitochondria (Perelman et al., 2012). Mitochondrial dysfunctions have been associated with various disorders, and a decrease in the mitochondrial membrane potential may also be linked to apoptosis. Sought to determine whether NE induces mitochondrial disruption in chondrocytes, we used flow cytometry analysis with JC-1 staining. As shown in Figures 4E,F, NE increased mitochondrial membrane depolarization, which caused a decrease in the red/green ratio in JC-1 dye-stained chondrocytes. Collectively, our data clearly show that NE induces chondrocytes apoptosis through a mitochondrial-dependent process.

### NE Induces Excess Intracellular Levels of ROS in Chondrocytes

**FIGURE 5:**
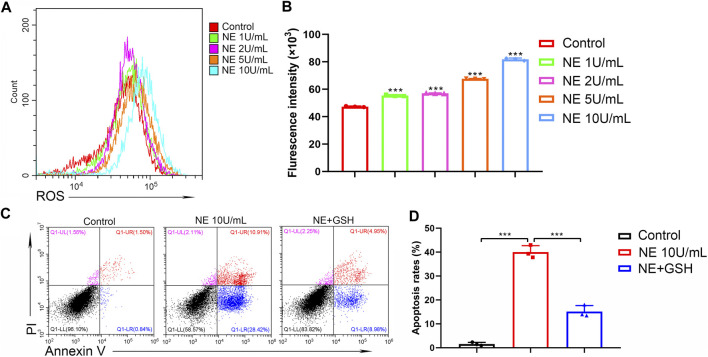
Neutrophil elastase induces ROS production of chondrocytes. **(A,B)** ROS levels of chondrocytes were measured by flow cytometry. **(A)** Representative histograms were shown. **(B)** Cell associated mean relative fluorescence intensities. Data are mean ± SEM; *n* = 3. ***p < 0.001. **(C,D)** Chondrocytes were treated with NE and GSH for 48 h and then stained with annexin V-FITC/PI followed by flow cytometry analysis. **(C)** The fluorescence pattern of annexin V-FITC/PI staining chondrocytes. **(D)** Percentages of annexin V positive cells for chondrocytes treated with NE and GSH. Data are mean ± SEM; *n* = 3. ***p < 0.001.

It has been considered that aberrant ROS production participates in the mechanism of cell apoptosis. In the process of cartilage physiology, ROS acts as an important mediator to maintain cartilage homeostasis and regulate chondrocyte differentiation. Moderate oxidative stress can act as an adaptive protective mechanism in chondrocytes; however, excessive oxidative stress may induce chondrocytes apoptosis. The intracellular ROS levels were measured by the fluorescence of 2′,7′-dichlorofluorescein diacetate (DCFDA) using flow cytometric analysis. As shown in Figures 5A,B, NE significantly elevated intracellular ROS levels of chondrocytes in a dose-dependent manner. Then chondrocytes were treated with glutathione (GSH, 2 mM), the key antioxidant in tissues which can scavenge intracellular ROS. The results in Figures 5C,D indicated that GSH can protect the chondrocytes from apoptosis induced by NE. Collectively, these data suggested that the NE-induced chondrocytes apoptosis is associated with excess intracellular ROS production.

### NE Inhibits the Motility of Chondrocytes

**FIGURE 6:**
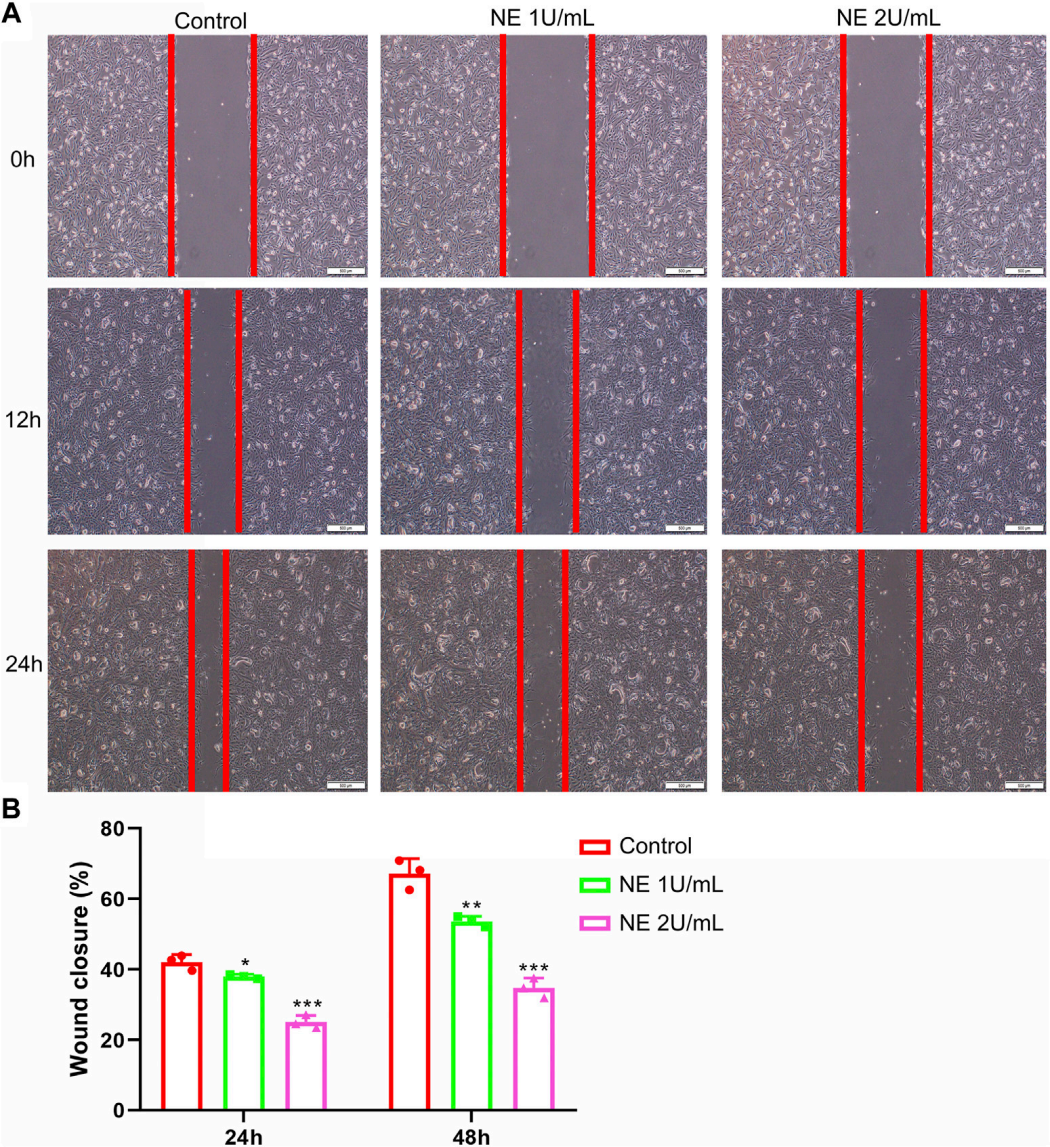
Neutrophil elastase inhibits the motility of chondrocytes. **(A,B)** Migration of chondrocytes was evaluated using the *in vitro* wound-healing scratch assay. **(A)** Representative photomicrographs of chondrocytes. **(B)** For quantitative analysis, percentage of wound closure was determined. Data are mean ± SEM; *n* = 3. *p < 0.05, **p < 0.01, ***p < 0.001.

Many cells use actin polymerization for directional migration or chemotaxis to soluble attractants. The phenomenon of directed cell migration plays an important role in embryonic morphogenesis, wound healing, inflammatory response and many disease states including cancer metastasis (Franz et al., 2002). Additionally, a wound-healing assay was performed to explore the effect of NE on the capability of chondrocytes to repair injury. The results in Figure 6 showed that NE prevented the repair of cell scratch injury in a concentration-dependent manner. Taken together, the assay revealed that NE repressed wound healing ability of chondrocytes, which suggested that blocking of NE might protect cartilage damage in OA.

### NE Induces Cell Retraction and Disrupts Actin Cytoskeleton of Chondrocytes

Confocal microscopy provides high contrast and resolution imaging which can display the detailed change in cell shape. In our study these changes were further detected by confocal microscope staining with phalloidin and DAPI: the nuclei are labeled with DAPI (in blue), and F-actin is labeled with phalloidin (in red). The cytoskeleton of eukaryotic cell consists of various filamentous structures, including intermediate filaments, actin filaments, and microtubules. Actin is a class of cytoskeletal proteins that assist cell motility and migration (Wear et al., 2000). Changes in actin architecture and dynamics can alter cell morphology, impair cell migration thus play a role in cell apoptosis. Hence, we accessed the formation of actin cytoskeleton in chondrocytes by staining with TRITC-phalloidin, a mushroom-derived fluorescent toxin, which is used to label F-actin of the cytoskeleton.

**FIGURE 7:**
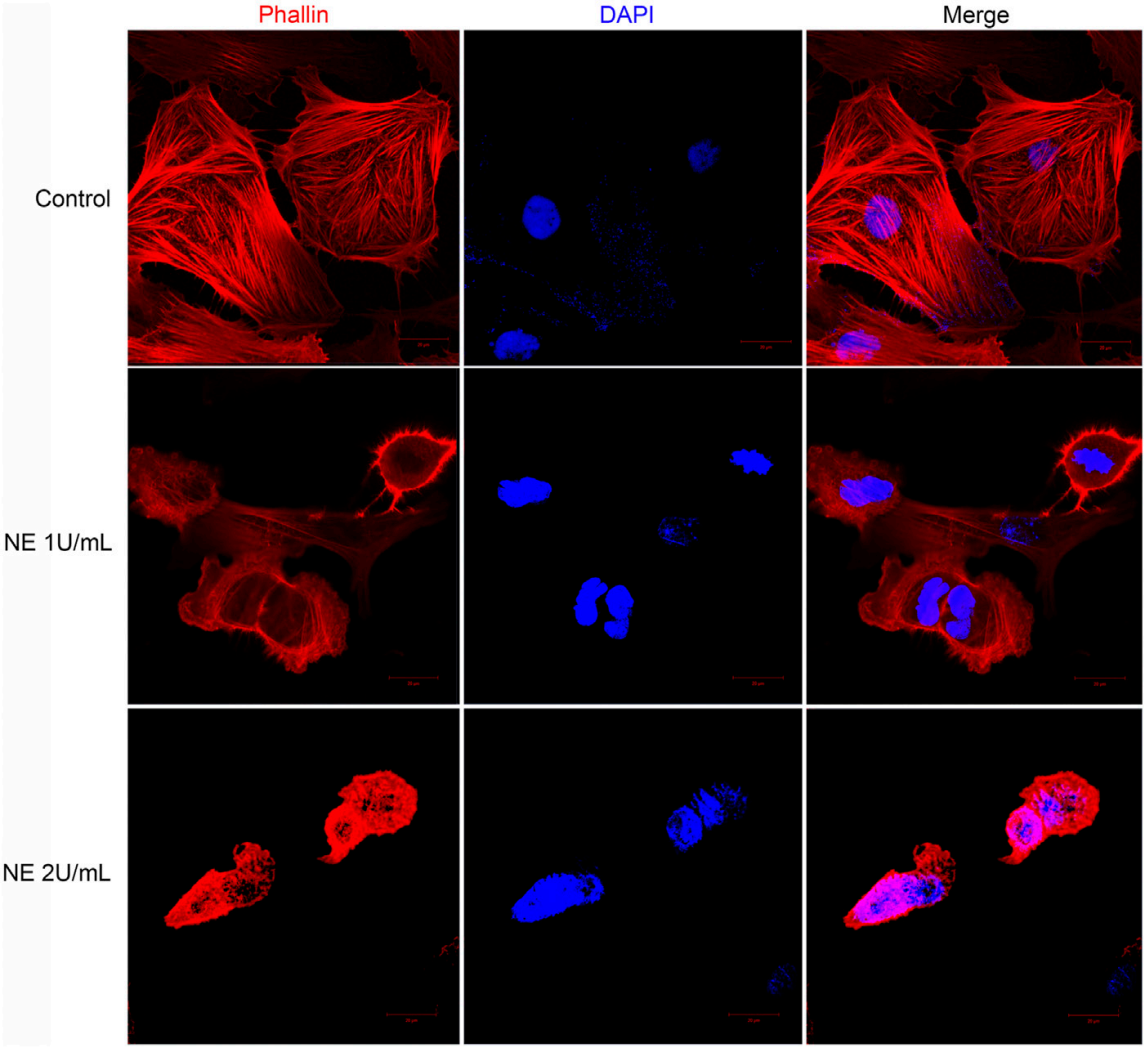
Neutrophil elastase induces retraction of chondrocytes and disrupts actin cytoskeleton. Representative confocal photomicrographs of cytoskeleton on chondrocytes treated with different dose of NE. Cells were fixed and stained with TRITC-phalloidin (red) and nuclei were stained with DAPI (blue). Original magnification, ×630.

As shown in Figure 7, F-actin assembly in chondrocytes was disrupted after NE treatment. For microscopic observations, compared with control groups, the size of the NE-treated cells was reduced and the distribution of actin was modified. All these observations support the fact that NE disorganizes the normal cytoskeleton in chondrocytes.

### Inhibition of NE in Cartilage Delays the Progression of OA in Rat Model

**FIGURE 8:**
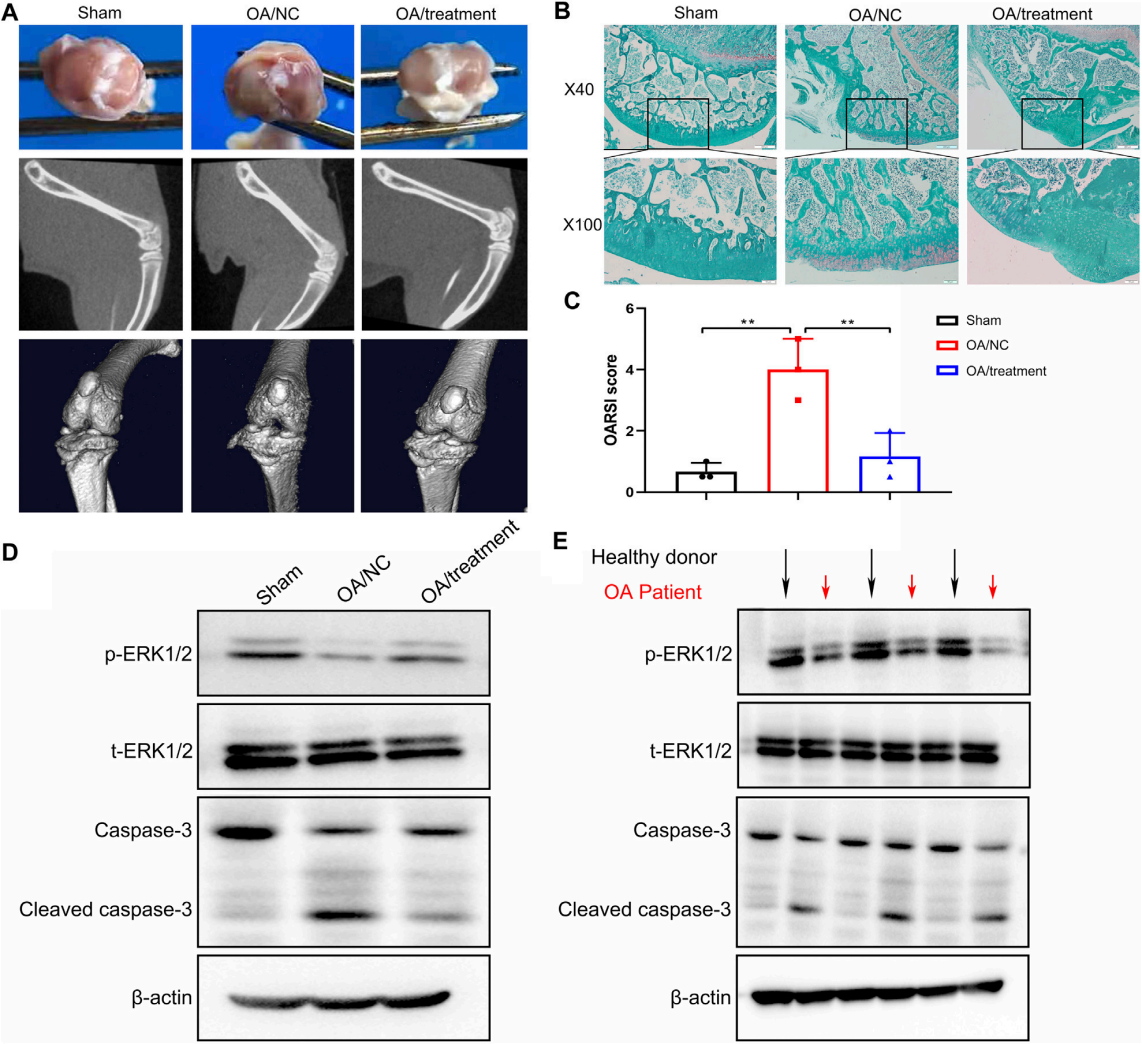
Sivelestat decreased apoptosis of articular chondrocytes and impeded the loss of cartilage in the knee joint from neutrophil elastase induced rat osteoarthritic model. **(A)** Macroscopic view, micro-CT image and 3D image of the specimens from sham group, OA model group and treatment groups. **(B)** Representative safranin O-fast green staining of the knee joint in sham group, OA model group and treatment groups. **(C)** OARSI scores based on staining results. Data are mean ± SEM; *n* = 3. **p < 0.01. **(D)** Western blot of ERK1/2 phosphorylation, total ERK1/2, caspase-3 and cleaved caspase-3 levels of articular cartilage from rat model. **(E)** Western blot of ERK1/2 phosphorylation, total ERK1/2, caspase-3 and cleaved caspase-3 levels of articular cartilage from healthy donors and OA patients.

From our *in vitro* study, we hypothesized that inhibition of NE in cartilage can delay the progression of OA *in vivo*. To test this hypothesis, an OA model were established in rats, followed by intraperitoneal injection of sivelestat for 2 weeks. Imaging investigations are useful to establish the severity of joint damage and monitor OA progression. As shown in Figure 8A, the macroscopic evaluation of cartilage surfaces indicated that control group had a smooth cartilage surface of knee joints, compared with obvious osteophytes formation and thickened surfaces observed in the knees of OA model. However, treatment with sivelestat, the inhibitor of NE, led to markedly less osteophytes formation without obvious surface thickening and reduced pathological processes in macroscopic images. Micro-CT was used to investigate the structure and density of bone in different groups, due to its high spatial resolution and high contrast in imaging mineralized tissues. Our micro-CT results revealed that OA model group showed obvious defect of cartilage surface and joint space narrowing compared to the sham group, and the sivelestat treatment abrogated the effect of NE.

We determined the impact of sivelestat on NE-induced OA by evaluating the structural features of articular cartilage with Safranin O staining and OARSI scores. The results of safranin O-fast green (S-O) staining (Figure 8B) showed that the surface of cartilage in knee joint was smooth and stained red in sham group. While in the OA group, we observed severe damage of morphological structure, cartilage erosion and lesions, remarkable cellular and proteoglycan loss. Nevertheless, sivelestat treatment abrogated the loss of chondrocytes and relieved the progression of OA. Concurrently, we found that the histological examinations were consistent with the Osteoarthritis Research Society International (OARSI) scores. Compared with sham controls, OARSI scores were significantly higher in OA group, and the rise was reduced significantly with sivelestat treatment (Figure 8C).

To elucidate the intrinsic mechanisms underlying the pro-apoptotic effects of NE *in vivo*, western blot analysis was performed to examine apoptosis-related markers. As shown in Figure 8D, the results implicated that NE treatment led to apparent increase in cleaved caspase-3 levels, and a significant decrease in the p-ERK1/2 levels in rat cartilage tissue, while these effects were weakened following sivelestat treatment. Similarly, we measured the levels of apoptosis-related proteins in the human specimen and the results showed that the articular cartilage in OA patients had low-level of p-ERK1/2 and high-level of cleaved caspase-3, which is coincide with the result of animal experiment (Figure 8E). Accordingly, our data indicated that inhibition of NE protected the cartilage from degradation and decreased the amounts of chondrocytes in the process of OA.

## Discussion

According to the current understanding, OA is a chronic progressive joint disease characterized by degenerative lesions of articular cartilage, reactive hyperplasia of articular edge and subchondral bone, and reduction of joint space. In clinical practice, OA conventional treatments like painkillers and non-steroidal anti-inflammatory drugs can only alleviate the state but do not reverse the disease. Recently the researchers focus on the tissue engineering therapies using biofunctionalized biomaterial to induce tissue repair and development, which is considered to be a novel strategy to restore joint structure and function. Due to the lack of effective drug treatment methods to delay disease progression or cure the OA, the research and development of new therapeutic targets for OA is a hot issue. Nowadays, accumulating studies have indicated that chondrocyte apoptosis was an early event in OA progress, and may play a key role in OA articular cartilage destruction by affecting a series of downstream pathophysiological processes (Hwang and Kim, 2015; Wei and Bai, 2016). Therefore, chondrocyte apoptosis pathway may be a potential therapeutic target for OA through drug intervention.

NE is a serine protease, which is abundant in mature neutrophils and mainly stored in the neutrophils of blue granules. With the development of research, researchers have gradually realized that NE has a variety of functions. NE may be a key regulator to promote the emergency myelopoiesis, leukocyte migration and homing, and can enhance the inflammatory response. OA is a transient inflammatory response in the early stages which is connected with high level of NE in the joint (Muley et al., 2017). In this work, we demonstrated that chondrocyte apoptosis was induced by NE. This study confirmed for the first time that the concentration of NE in the synovial fluid of patients with OA was significantly higher than that of healthy donors, suggesting that NE may be a key factor in the pathophysiological process of OA.

*In vitro* cell experiment, in order to explore the effect of NE on chondrocytes in the pathogenesis of OA, we first detected the effect of NE on the proliferation, apoptosis and migration of chondrocytes. It was found that NE significantly inhibited cell proliferation, promoted chondrocyte apoptosis and prevented the repair of cell scratch injury.

Furthermore, we demonstrated whether NE promotes the intrinsic apoptotic pathway of chondrocytes from OA progression. It has been previously reported that the intrinsic apoptotic signaling pathway can be caused by a variety of intracellular damages, including DNA fragmentation, oxidative stress damage and intracellular calcium imbalance. These damage signals will eventually change the permeability of mitochondrial outer membrane permeabilization (MOMP), leading to the leakage of a variety of substances from mitochondria into the cytoplasm, such as cytochrome c (cyt c), and finally activate the mitochondrial caspase pathway. Consistent with the theory, our study further confirmed that NE can induce chondrocytes apoptosis through intracellular ROS production, DNA degradation and cytosolic calcium elevation, thus causing mitochondrial membrane potential disruption.

Caspase-3 can activate a variety of apoptosis related substrates and then induce apoptosis. Inhibiting caspase-3 activation in a variety of ways may be an important strategy to reduce chondrocyte apoptosis. In our work, western blot and flow cytometry were used to analyze the specific signal transduction process of NE induced chondrocyte apoptosis. Our data of cell experiments, animal experiments and clinical samples show that NE can induce chondrocyte apoptosis by regulating a variety of apoptosis related signaling molecules. In addition, our *in vitro* studies exhibited that the Z-VAD-FMK, the caspase inhibitor which was used to inhibit apoptosis controlled by caspase, significantly suppressed chondrocyte apoptosis.

Animal model is an important tool to study the mechanisms and effect *in vivo*. In order to explore the specific mechanism of NE induced OA *in vivo*, we try to build a rat OA model based on our theoretical hypothesis. Therefore, we established an experimental rat OA model by intra-articular injection of NE to simulate the OA observed in clinical practice. In the OA rat model, the gross pathological manifestations include severe destruction and local shedding of cartilage on the surface of knee joint, and the histopathological manifestations include significant reduction of chondrocytes and thickness of extracellular matrix. The micro-CT data clearly show that NE can significantly reduce the knee joint space in rats, and the articular surface is fuzzy and unsmooth. Sivelestat, an inhibitor of NE, can significantly relieve the symptoms of OA induced by NE and reduce the apoptosis of chondrocytes. The whole data indicate that NE is the key factor to induce OA in the body, and inhibiting the function of NE can significantly prevent the pathophysiological process of OA.

## Conclusion

In summary, our present study demonstrated that NE could promote chondrocyte apoptosis and facilitate the occurrence of OA *via* caspase-3 signalling pathway *in vitro* and *in vivo*. Meanwhile, the therapeutic efficacy of NE inhibitor was also confirmed *in vivo*. These results underscore an intriguing role for NE as a crucial target in the treatment of OA.

## Data Availability

The original contributions presented in the study are included in the article/Supplementary Material, further inquiries can be directed to the corresponding authors.
